# Suicide epidemics: the impact of newly emerging methods on overall suicide rates - a time trends study

**DOI:** 10.1186/1471-2458-11-314

**Published:** 2011-05-14

**Authors:** Kyla Thomas, Shu-Sen Chang, David Gunnell

**Affiliations:** 1 School of Social and Community Medicine, University of Bristol, Bristol, UK; 2 Ju Shan Hospital, Taoyuan, Taiwan

## Abstract

**Background:**

The impact of newly emerging, popular suicide methods on overall rates of suicide has not previously been investigated systematically. Understanding these effects may have important implications for public health surveillance. We examine the emergence of three novel methods of suicide by gassing in the 20^th ^and 21^st ^centuries and determine the impact of emerging methods on overall suicide rates.

**Methods:**

We studied the epidemic rises in domestic coal gas (1919-1935, England and Wales), motor vehicle exhaust gas (1975-1992, England and Wales) and barbecue charcoal gas (1999-2006, Taiwan) suicide using Poisson and joinpoint regression models. Joinpoint regression uses contiguous linear segments and join points (points at which trends change) to describe trends in incidence.

**Results:**

Epidemic increases in the use of new methods of suicide were generally associated with rises in overall suicide rates of between 23% and 71%. The recent epidemic of barbecue charcoal suicides in Taiwan was associated with the largest rise in overall rates (40-50% annual rise), whereas the smallest rise was seen for car exhaust gassing in England and Wales (7% annual rise). Joinpoint analyses were only feasible for car exhaust and charcoal burning suicides; these suggested an impact of the emergence of car exhaust suicides on overall suicide rates in both sexes in England and Wales. However there was no statistical evidence of a change in the already increasing overall suicide trends when charcoal burning suicides emerged in Taiwan, possibly due to the concurrent economic recession.

**Conclusions:**

Rapid rises in the use of new sources of gas for suicide were generally associated with increases in overall suicide rates. Suicide prevention strategies should include strengthening local and national surveillance for early detection of novel suicide methods and implementation of effective media guidelines and other appropriate interventions to limit the spread of new methods.

## Background

Suicide is among the leading causes of premature mortality in the world [1]. In 2004 suicide was the 8^th ^leading cause of potential years of life lost among 15 to 44 year olds worldwide [1]. Whilst social, economic, cultural and psychological factors are significant contributors to suicide rates, there is good evidence that the changing availability and popularity of lethal methods are also important [2-4].

To date, most studies investigating associations between method availability and suicide rates have focused on the impact of limiting access to methods, such as the detoxification of the domestic gas supply in the 1950s and 1960s, the introduction of catalytic converters reducing the toxicity of car exhausts in the 1990s and the withdrawal of co-proxamol in recent years in the UK [5,6]. By the time these restrictions are put in place, many deaths may have already been caused by a particular method. Little attention has been paid to the impact of newly emerging methods on suicide rates, although the recent epidemics of charcoal burning suicides in Hong Kong and Taiwan have been well documented [7]. The rapid rise in the popularity of charcoal burning in the Far East is thought to have been fuelled by the extensive media and internet coverage of suicides using this method [8]. However much less is known about the reporting of domestic gas and car exhaust gassing suicides during rapid increases in the use of these methods in England and Wales.

The aim of this analysis is to compare the rate of increase of suicide by gassing that occurred during three periods of rapidly rising use of this method in two countries - Taiwan and England and Wales at three different time periods in the 20^th ^and 21^st ^centuries. Different sources of gas were used in each epidemic - domestic ("coal") gas (1920s and 1930s in England and Wales), motor vehicle exhaust gas (1970s and 1980s in England and Wales) and barbecue charcoal gas (Taiwan 2000s). We assess whether the emergence of new methods leads to an increase in overall suicide rates. We also hypothesize that the speed of uptake of novel methods may have been lower before the widespread availability of television and the Internet. The first epidemic (coal gassing suicides in England and Wales) occurred before the widespread availability of television, the second (car exhaust gassing in England and Wales), before the widespread popularity of the Internet whereas the epidemic of barbecue charcoal burning suicides in Taiwan occurred after both types of media had become widely available.

## Methods

Sex-specific suicide mortality and population data for England and Wales between 1911-2007, by year and in 5 year age bands, were obtained from the Office for National Statistics. Similar data for Taiwan were provided by the Department of Health, Taiwan and mid-year population estimates were obtained from the Demographic Yearbook, published by the Ministry of the Interior. Gassing suicides for England and Wales were identified from the International Classification of Diseases (ICD) codes as follows (ICD-2 156, ICD-3 167, ICD-4 164, from 1919 to 1935, ICD-8 and ICD-9 E951-E952 from 1975-1992). For Taiwan, gassing suicides were also estimated based on E951 and E952 cases (1999-2006). Non-gassing suicides were determined using the following codes excluding the gassing suicide codes as previously defined (ICD-2 150-163, ICD-3 165-174, ICD-4 163-171, ICD-8 and ICD-9 E950-E959). The category 'deaths undetermined whether deliberate or accidental' was included from the 8^th ^revision of the ICD (ICD-8) in 1968 as up to 75% of these deaths are probable suicides [9]. Gas deaths are coded as E981-E982 and deaths by other methods as E980-E988 excluding the previously defined codes.

**Figure 1:**
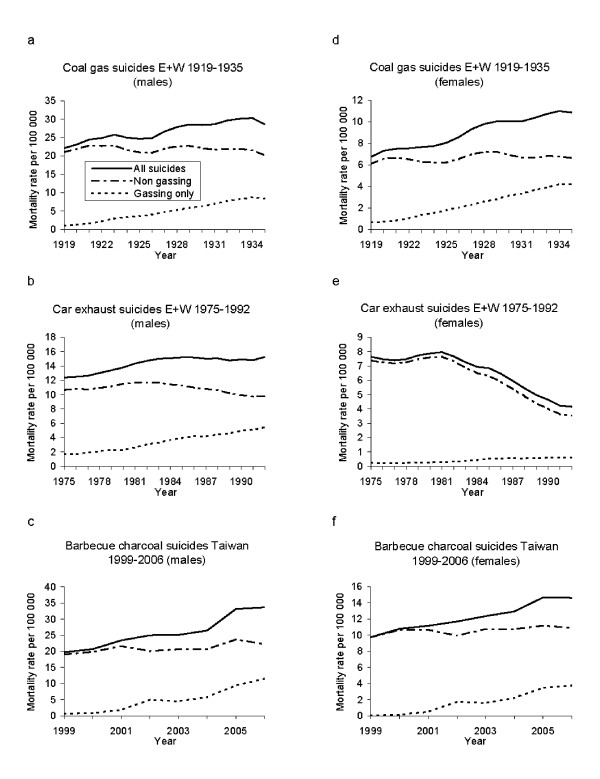
**Trends in age-standardized suicide rates for males and females in England and Wales (E+W) and Taiwan over 3 time periods**.

Yearly age standardized rates were calculated for ages 15 and over using the European Standard population. We selected the years for the Poisson regression analysis based on visual inspection of graphs of trends in rates of gassing suicides (see Figure 1) complemented with findings from the joinpoint analysis for car exhaust and charcoal burning epidemics only (described in detail below). The time periods were as follows:

1. 1919-1935 domestic coal gas poisoning (England and Wales).

2. 1975-1992 motor vehicles exhaust gas suicides (England and Wales).

3. 1999-2006 barbecue charcoal gas suicides (Taiwan).

Poisson regression analyses were used to estimate the annual rate of change in method-specific and overall suicide rates using the rate in the first year of each period studied as the reference category. These models assume a linear increase in rates. Incidence rate ratios for gassing, non-gassing and overall suicides were calculated by sex for ages 15 and over (all ages) and three age groups (15-44, 45-64 and 65 and over). The calculations were repeated including undetermined deaths for the time periods 1975 to 1992 (England and Wales) and 1999-2006 (Taiwan). Since the category 'undetermined death' was not introduced until 1968 this analysis was not possible for data on domestic gas poisoning in England and Wales in the 1920s and 1930s. We included interaction terms between sex and year as well as age group and year to assess whether the annual rate of increase in suicides varied between sexes or among people of different age groups.

We used joinpoint regression to further investigate the impact of rises in car exhaust and charcoal burning suicides on longer term trends in suicide rates in England and Wales (1966-1992) and Taiwan (1987-2007). The impact of coal gas suicides on overall suicide rates in England (1919-1935) was not assessed as the incidence of suicide for the years preceding the epidemic rise was influenced by the impact of the First World War (1914-1918) on rates [3]. Joinpoint regression uses contiguous linear segments and join points (points at which trends change) to describe trends in incidence. Suicide rates in the periods prior to the emergence of new suicide methods were used as indicators of pre-existing underlying trends. We used a 10 year period for trends in England and Wales, but a longer period for Taiwan to capture Taiwan's falling suicide rates prior to 1992. The number and location of join points were detected and annual percent changes in rates between join points were estimated based on linear regression. A sequence of permutation tests were used when comparing pairs of models differing by one join point. The overall probability of type 1 error (i.e. concluding that there is one or more join points when there are in fact none) was maintained at 0.05. The number of join points was limited to a maximum of three. Analyses were carried out using the Joinpoint software (version 3.2, National Cancer Institute Bethesda, MD). For the majority of models log rate was used as the dependent variable. However raw rate was used in the model for Taiwan as the original model did not correctly describe the acute rise in the gassing suicide rate. Additionally, joinpoint regression could not be performed on data for Taiwanese females due to the presence of zero values for some of the years.

## Results

Figure 1 shows the sex-specific suicide rates for overall, gassing and non-gassing suicides over the three periods when the use of gassing as a method of suicide increased. There were 17 676 gassing suicides from 1919 to 1935 (1040 per year) and 13 806 from 1975 to 1992 in England and Wales (767 per year); 5463 barbecue charcoal deaths occurred in Taiwan from 1999 to 2006 (683 per year).

Overall suicide rates increased in 5 out of the 6 plots. The increase in each time period was almost entirely due to the increase in suicides by gassing; the one exception was for females in England and Wales from 1975 to 1992 (Figure 1 and Table 1). Although gassing suicides tripled in incidence in females during this period, the rate was only 0.2 per 100 000 at the beginning of the epidemic; therefore the absolute increase in rates was very small (0.4 per 100 000). Suicides by all other methods decreased by 52.8% over the period studied.

**Table 1:** Age standardized suicide rates by sex in England and Wales (E+W) and Taiwan for the three time periods

	Time period	Method	Rates per 100 000 at beginning of analytic period	Rates per 100 000 at end of analytic period	Difference in rates per 100 000	% change
MALES	1919-1935 E+W					
		*All suicides*	22.1	28.6	+6.5	+ 29.4
		*Non-gassing suicides*	21.0	20.1	-0.9	-4.3
		*Gassing suicides*	1.1	8.5	+7.4	+672.7
	1975-1992 E+W					
		*All suicides*	12.4	15.2	+2.8	+22.6
		*Non-gassing suicides*	10.7	9.8	-0.9	-8.4
		*Gassing suicides*	1.8	5.5	+3.7	+205.6
	1999-2006 Taiwan					
		*All suicides*	19.7	33.7	+14.0	+71.1
		*Non-gassing suicides*	19.1	22.2	+3.1	+16.2
		*Gassing suicides*	0.6	11.5	+10.9	> 1000
FEMALES	1919-1935 E+W					
		*All suicides*	6.7	10.8	+4.1	+61.2
		*Non-gassing suicides*	6.1	6.6	+0.5	+8.2
		*Gassing suicides*	0.6	4.2	+3.6	+600
	1975-1992 E+W					
		*All suicides*	7.6	4.1	-3.5	-46.1
		*Non-gassing suicides*	7.4	3.5	-3.9	-52.8
		*Gassing suicides*	0.2	0.6	+0.4	+200
	1999-2006 Taiwan					
		*All suicides*	9.7	14.7	+5.0	+51.5
		*Non-gassing suicides*	9.7	11.0	+1.3	+13.4
		*Gassing suicides*	0.0	3.7	+3.7	> 1000
BOTH SEXES	1919-1935 E+W					
		*All suicides*	14.9	17.8	+2.9	+19.5
		*Non-gassing suicides*	14.0	11.8	-2.2	-15.7
		*Gassing suicides*	0.9	6.0	+5.1	+566.7
	1975-1992 E+W					
		*All suicides*	9.6	9.6	+0.0	0.0
		*Non-gassing suicides*	8.8	6.5	-2.3	-26.1
		*Gassing suicides*	0.8	3.1	+2.3	+287.5
	1999-2006 Taiwan					
		*All suicides*	14.8	24.2	+9.4	+63.5
		*Non-gassing suicides*	14.5	16.6	+2.1	+14.5
		*Gassing suicides*	0.3	7.6	+7.3	> 1000

In males, overall suicide rates increased by 29.4% from 1919 to 1935 and by 22.6% from 1975 to 1992 in England and Wales; rates increased by 71.1% from 1999 to 2006 in Taiwan (Table 1). Gassing suicides were uncommon in England and Wales before 1919 but rose to account for 29.7% of all male suicides by 1935. Gassing suicides, as a percentage of all male suicides increased from 14.5% to 36.2% between 1975 and 1992 in England and Wales and from 3.1% to 33.4% between 1999 and 2006 in Taiwan.

In females overall suicide rates increased by 61% from 1919 to 1935, but decreased by 46% from 1975 to 1992 in England and Wales (see Table 1). An increase of 51.5% was seen from 1999 to 2006 in Taiwan. Gassing accounted for 8.9% of all female suicides in 1919 but by 1935 this figure had risen to 38.9%. The percentage of gassing suicides increased from 2.6% to only 14.6% of all female suicides between 1975 and 1992 in England and Wales and from 0.2% to 24.2% from 1999 to 2006 in Taiwan.

### Time trends analyses

Table 2 shows the annual rate of change in suicide rates in males and females of all ages and by age group. For males of all ages, the yearly increase in the incidence of gassing suicides was lowest for car exhaust gassing in England and Wales from 1975 to 1992 (7.2%, 95% CI 6.8%-7.6%), and highest for charcoal burning suicides in Taiwan (40.1%, 95% CI 37.9%-42.3%) from 1999 to 2006. In males there was a smaller yearly increase in overall suicide rates in England and Wales 1.7%, (95% CI 1.5%-1.9%) from 1919-1935 and 1.2%, (95% CI 1.0%-1.4%) from 1975-1992, compared with Taiwan 7.5% (95% CI 6.9%-8.2%) from 1999-2006. Additionally there was a yearly decrease in the suicide incidence rate for non-gassing suicides in England and Wales of 0.3% (95% CI 0.2%-0.5%) from 1919 to 1935 and 0.7% (95% CI 0.5%-0.9%) from 1975 to 1992 indicating some possible method substitution. Such an effect was not seen in Taiwan. These findings were broadly similar in the age-specific analyses although the relative rises in overall suicide rates in Taiwan were greater in younger compared with older men.

**Table 2:** Incidence rate ratios for suicide and 95% confidence intervals by sex for all ages and 3 age groups

			Incidence rate ratios (95% CI)		
			Coal gas England and Wales (1919-1935)	Car exhaust gas England and Wales (1975-1992)	Barbecue charcoal gas Taiwan (1999-2006)
**ALL AGES**					
	Males				
		*All suicides*	1.017 (1.015-1.019)	1.012 (1.010-1.014)	1.075 (1.069-1.082)
		*Non gassing*	0.997 (0.995-0.999)	0.993 (0.991-0.995)	1.019 (1.012-1.025)
		*Gassing only*	1.106 (1.101-1.110)	1.072 (1.068-1.076)	1.401 (1.379-1.423)
	Females				
		*All suicides*	1.032 (1.029-1.034)	0.959 (0.957-0.962)	1.053 (1.044-1.062)
		*Non gassing*	1.005 (1.002-1.008)	0.952 (0.950-0.955)	1.011 (1.001-1.020)
		*Gassing only*	1.108 (1.102-1.114)	1.071 (1.061-1.083)	1.469 (1.426-1.513)
**15-44 years**					
	Males				
		*All suicides*	1.014 (1.011-1.017)	1.032 (1.029-1.034)	1.100 (1.091-1.110)
		*Non gassing*	0.992 (0.989-0.995)	1.012 (1.009-1.015)	1.017 (1.007-1.027)
		*Gassing only*	1.105 (1.097-1.112)	1.079 (1.074-1.084)	1.434 (1.406-1.463)
	Females				
		*All suicides*	1.030 (1.026-1.034)	0.972 (0.967-0.976)	1.070 (1.057-1.084)
		*Non gassing*	1.006 (1.002-1.011)	0.959 (0.954-0.963)	0.997 (0.982-1.011)
		*Gassing only*	1.108 (1.099-1.118)	1.087 (1.072-1.103)	1.461 (1.413-1.511)
**45-64 years**					
	Males				
		*All suicides*	1.018 (1.015-1.020)	0.998 (0.995-1.001)	1.060 (1.049-1.072)
		*Non gassing*	0.998 (0.996-1.001)	0.978 (0.975-0.982)	1.016 (1.005-1.028)
		*Gassing only*	1.101 (1.095-1.108)	1.063 (1.056-1.070)	1.399 (1.355-1.445)
	Females				
		*All suicides*	1.026 (1.022-1.030)	0.957 (0.954-0.961)	1.034 (1.018-1.051)
		*Non gassing*	0.998 (0.993-1.003)	0.951 (0.947-0.955)	0.997 (0.980-1.013)
		*Gassing only*	1.099 (1.090-1.107)	1.060 (1.042-1.077)	1.544 (1.446-1.648)
**65+ years**					
	Males				
		*All suicides*	1.010 (1.006-1.014)	0.989 (0.985-0.993)	1.020 (1.007-1.033)
		*Non gassing*	0.991 (0.986-0.995)	0.980 (0.976-0.984)	1.013 (1.000-1.026)
		*Gassing only*	1.109 (1.097-1.121	1.065 (1.052-1.078)	1.536 (1.369-1.723)
	Females				
		*All suicides*	1.035 (1.026-1.043)	0.951 (0.947-0.955)	1.014 (0.997-1.032)
		*Non gassing*	1.006(0.997-1.015)	0.950 (0.945-0.954)	1.012 (0.995-1.029)
		*Gassing only*	1.114 (1.097-1.133)	1.059 (1.022-1.098)	1.415 (1.109-1.804)

In females of all ages the relative rises in gassing suicides in all three eras were similar to those seen in males. However, despite the rise in suicide by car exhaust gassing from 1975 to 1992 in females, overall suicide rates decreased yearly by 4.1% (95% CI 3.8%-4.3%).

There was evidence that the rates of increase in barbecue charcoal suicides were higher in Taiwanese females than males aged 45-64 years (p for interaction < 0.0072). There was no statistical evidence that rates of uptake of gassing differed in the three age groups studied. Inclusion of undetermined deaths in the Poisson regression models had little impact on the incidence rate ratios.

### Joinpoint regression analyses (England and Wales 1966-1992 and Taiwan 1987-2007)

**Figure 2:**
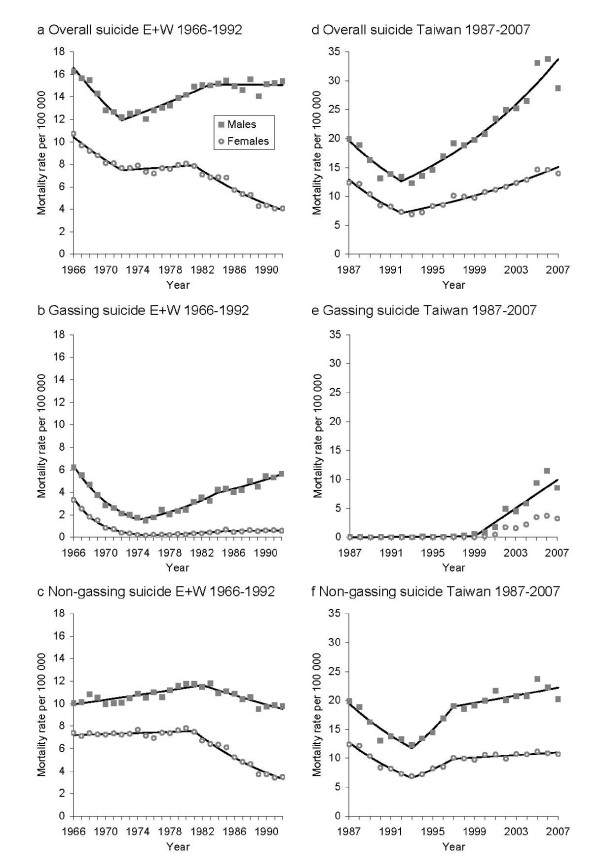
**Joinpoint regression analyses of trends in age-standardized suicide rates by sex for England and Wales (E+W) 1966-1992 and Taiwan 1987-2007 (analyses not performed for Taiwanese females due to zero values for some of the years)**.

In England and Wales downward trends in overall suicide rates in the late 1960s changed around 1972 (95% CI 1971-1975 for males, 1969-1977 for females) when male rates began to rise (annual percent changes [APCs] 2.2%, 95% CI 1.4%-2.9%) and female rates became relatively stable (APCs 0.6%, 95% CI -0.8%-2.0%) (see Table 3 and Figure 2a). The timing of these changes corresponds to the rise in car exhaust gassing and there was no clear evidence of method substitution (Table 3, Figures 2b and 2c).

**Table 3:** Summary of the annual percent changes (APCs) and joinpoints (JPs) for trends in age-standardized suicide rates by sex for England and Wales (1966-1992) and Taiwan (1987-2007)

	Segment 1		Segment 2		Segment 3
					
	APC (95% CI)	JP 1 (95% CI)	APC (95% CI)	JP 2 (95% CI)	APC (95% CI)
Overall suicide					
*England and Wales (2 join points for males and one join point for females)*					
Male	-5.4 (-6.8, -3.9)	1972 (1971, 1975)	2.2 (1.4, 2.9)	1983 (1979, 1986)	0.0 (-0.8, 0.7)
Female	-5.4 (-7.2, -3.5)	1972 (1969, 1977)	0.6 (-0.8, 2.0)	1981 (1979, 1985)	-6.2 (-7.1, -5.2)
*Taiwan (1 join point for males and females)*					
Male	-8.5 (-15.0, -1.5)	1992 (1989, 1994)	6.8 (5.7, 7.9)		
Female	-11.2 (-15.0, -7.2)	1992 (1991, 1994)	5.1 (4.4, 5.8)		
Gassing suicide					
*England and Wales (2 join points for males and one join point for females)*					
Male	-16.1 (-18.3, -13.9)	1974 (1973, 1976)	9.8 (7.0, 12.7)	1984 (1979, 1988)	4.4 (2.3, 6.7)
Female	-27.9 (-29.6, -26.2)	1975 (1974, 1976)	12.0 (7.5, 16.6)	1985 (1982, 1989)	1.3 (-3.1, 5.8)
Taiwan (1 join point for males*; join point regression not performed for females due to zero values for some of the years)					
Male*	0.01 (-0.003, 0.03)	1999 (1998, 2001)	1.2 (1.0, 1.4)		
Female	-				
Non-gassing suicide					
*England and Wales (1 join point for males and females)*					
Male	1.0 (0.6, 1.4)	1982 (1980, 1984)	-2.0 (-2.7, -1.3)		
Female	0.3 (-0.1, 0.8)	1981 (1980, 1983)	-7.2 (-8.1, -6.4)		
*Taiwan (2 join points for males and females)*					
Male	-8.0 (-11.7, -4.2)	1993 (1989, 1995)	12.7 (0.7, 26.1)	1997 (1995, 2005)	1.6 (0.2, 3.0)
Female	-10.4 (-12.5, -8.2)	1993 (1991, 1994)	10.7 (3.0, 18.9)	1997 (1996, 1999)	1.1 (0.2, 2.0)

*Annual change in rates per 100,000, not annual percent change, shown.

In contrast, overall suicide trends in Taiwan did not appear to change around the time when the epidemic of charcoal burning suicide emerged in Taiwanese men in 1999, 95% CI 1998-2001 (Table 3 and Figure 2d). Overall suicide trends fell in the late 1980s and early 1990s and began to increase from 1992 (95% CI 1989-1994 for males and 1991-1994 for females) with continuously upward trends over the mid to late 1990s and 2000s (APCs 6.8%, 95% CI 5.7%-7.9% for males and 5.1%, 95% CI 4.4%-5.8% for females). Increases in overall suicides between 1999 and 2006 were almost entirely due to barbecue charcoal suicides (see Figures 2d and 2e). The previously rapid rises in rates of non-gassing suicides in Taiwan from 1993 (95% CI 1989-1995 for males and 1991-1994 for females) leveled off from 1997 (95% CI 1995-2005 for males and 1996-1999 for females), two years before the beginning of the charcoal burning suicide epidemic in 1999 (see Table 3, Figures 2e and 2f). Therefore it is possible that the continuing increase in overall suicide rates observed in Taiwan from 1999 onwards was due to (a) "new" suicides associated with the emergence of charcoal burning as a method of suicide at a time when rates of increase in overall suicides would otherwise have leveled off or (b) a continuation of pre-existing upward trends, but with method substitution to charcoal burning from other non-gassing methods to the novel gassing method or (c) a combination of these two phenomena.

## Discussion

### Main findings

The three epidemics of gassing suicides were generally associated with increases in overall suicide rates, although the extent of the rises differed by era, sex and actual method. Overall, there were approximately 36 949 suicides associated with new methods of gassing; 1040 gassing suicides per year from 1919 to 1935 and 767 per year from 1975 to 1992 in England and Wales; 683 barbecue charcoal deaths per year occurred in Taiwan from 1999 to 2006. If we assume that 30% of these deaths might not have otherwise occurred had the methods not become popular (based on the approximate percentage reduction in suicides following coal gas detoxification in England and Wales [4]), then there would have been between 205 (Taiwan 1999-2006) and 312 (England and Wales 1919-1935) fewer suicides per year. The recent epidemic of barbecue charcoal burning suicides in Taiwan was associated with the largest rise in overall rates, whereas the rise in car exhaust gassing (England and Wales 1975-1992) had the smallest impact. Although the relative increase in rates of car exhaust gassing was similar in males and females the absolute increase in female gassing suicide rates involving this method was minimal (0.2 to 0.6 per 100 000 from 1975 to 1992). There was no conclusive evidence that the rate of uptake of novel gassing increased with successive epidemics. There was some evidence for the impact of the emergence of car exhaust gas suicide on overall rates in both men and women in England and Wales while there was no evidence of a change in underlying trends for overall suicides when Taiwan's epidemic of charcoal burning suicides began in 1999. However, it is not possible to determine from our data whether the increase in overall suicide rates in Taiwan after 1999 was due to an increase in suicide rates associated with the new method or a continuation of previously upward trends with method substitution from other non-gassing methods to charcoal burning. The observation that the timing of the changes in the rate of increase of non-gas suicide (in 1997) preceded the start of the epidemic of charcoal burning suicide (in 1999) by two years, makes method substitution a less likely explanation, although both mechanisms may have contributed to observed trends.

### Strengths and Limitations

This is the first study to compare the impact of emerging methods on overall suicide rates across a variety of settings and methods. Although good quality, national level data were obtained there are several limitations of this analysis. First, there were changes in the ICD classification used in the different time periods. However within each time period there were no obvious visual discontinuities in trends when there was a change in coding. Also, it has been shown that changes between ICD-6, ICD-7 and ICD-8 and ICD-9 did not have an effect on overall reported suicide rates [10]. Second, there may have been changes in the quality of reporting, affecting coding accuracy over time. A recent paper in Taiwan suggested increased misclassification of suicides as undetermined deaths in the 1990s [11]. It is reassuring that our analysis did not show any differences in findings when undetermined deaths were also included. Although increased awareness of a specific method may also lead to better reporting, the large magnitude of the rises in suicide rates seen in this study make it highly unlikely that the observed increases were due solely to improvements in reporting. Third, gassing suicides were not further differentiated into specific methods of gassing. We assumed that from 1919 to 1935, the majority of gassing suicides would have been due to domestic gas since cars were not widely available at that time; similarly from 1975 to 1992, we assumed that the majority of gassing suicides were due to motor vehicle exhaust gassing as domestic gas had been detoxified [4]. The majority of gassing suicides from 1999 to 2006 in Taiwan have been shown previously to be due to charcoal burning barbecue suicides [12]. Last, we did not adjust for other variables that may influence suicide rates such as economic recession, unemployment, levels of divorce and changes in alcohol consumption, although it is unlikely [13,14] that these factors would impact on gassing suicides only without affecting other methods.

### Findings in context of literature

Other suicide epidemics that have been identified from the literature include the epidemics of pesticide poisoning in Sri Lanka, Western Samoa and South Korea (1960s until 1995, 1970 to 1981 and 1996 to 2005 respectively), charcoal burning in Hong Kong (1998 to 2002) and self poisoning with seeds of the yellow oleander tree in Northern Sri Lanka (1980s to mid 1990s) [15-19]. Initially, the latter two methods were rarely observed within national suicide statistics. However extensive media reporting of a few cases was blamed for the subsequent increase in popularity of these methods. In Sri Lanka widespread local reporting of the suicides of two schoolgirls in Jaffna caused by eating yellow oleander seeds caused an increase in the number of cases seen in the local hospital from nil in 1980 to 23 in 1981, 46 in 1982 and 103 in 1983. The extensive reporting in November 1998 of the case of a 35 year-old woman who died by charcoal burning has also been blamed for the rapid emergence of this method in Hong Kong and its spread to other regions such as Taiwan and Japan [18]. As a result in Hong Kong the population suicide rate increased from 12 per 100 000 (prior to the reporting of the first charcoal burning case) to 18.6 per 100 000 in 2003. By 2003, barbecue charcoal burning was responsible for 25% of deaths from suicide in Hong Kong.

Unemployment was a key factor influencing Taiwan's suicide rates in the late 1980s and early 1990s [13]. The rise in unemployment in the 1990s and its effect on the incidence of suicide may have masked the early impact of the charcoal burning epidemic. This is supported by the observation that after unemployment rates reached a peak in 2002 and subsequently declined, suicide rates continued to increase, coinciding with the emergence of charcoal burning suicide [13]. In addition, method-specific trends showed that charcoal burning contributed to most of the rise in overall suicide rates after 1998-2000 [20].

Although we had hypothesized that there might have been a stepwise increase in uptake of new methods with successive epidemics, evidence for this was equivocal. The differences seen in uptake of barbecue charcoal gas versus other gassing methods may have been due to the greater influence of the media in the 1990s compared with the 1970s or 1920s. However we cannot rule out the impact of cultural differences between the UK and Taiwan. Furthermore, the rate of uptake of domestic gassing from 1919 to 1935 was greater than the uptake of car exhaust gassing 50 years later, although this difference may reflect the greater degree of planning and technical knowledge required to implement the latter method.

Gassing has been shown to be acceptable as a suicide method to men and women [21]. However its relative use by males and females may depend on the source of gas used. For example the male to female sex ratio for the rate of domestic (coal) gas suicide from the 1920s to 1950s was approximately 2:1; for the era when car exhaust gassing was more common the ratio was 8-9:1 in the late 1970s and 1980s [3]. It is possible that the differences in the preparation required for each type of gassing method were likely to contribute to these differences, with the increased technicality involved in car exhaust suicides and ease of access to cars acting as barriers to females.

### Public Health Implications

The results of this study have implications for suicide prevention strategies. Means restriction, i.e. limiting access to suicide methods has been shown in many previous population studies to be an effective approach to reducing suicides in the general population. This research indicates that it is also important to establish or strengthen suicide surveillance mechanisms at both local and national levels in order to identify newly emerging methods of suicide so that intervention can occur at the earliest possible stage to prevent increases in overall suicide rates. The media and internet have been identified as playing a crucial role in the dispersion of information about novel suicide methods [18]. In Vienna, media guidelines for the responsible reporting of suicides were introduced in 1987, banning newspapers from reporting the methods of suicides [22]. It was hypothesized that the 80% reduction in the rates of subway suicides observed in the subsequent 6 months was due to the introduction of these guidelines. A more recent study using interrupted time series analysis has confirmed these findings and the authors believed that the effects were due to changes in the quality and quantity of reporting [23]. However a delicate balance needs to be maintained between press freedom and responsibility of the press to minimize the harm to vulnerable individuals. The media can provide both negative as well as positive influences since it can also be used as a source of information about where to seek help and advice. A recent study has also described a 'Papageno' protective effect of media reporting, if the reports focus on individuals who adopt positive coping strategies in adverse circumstances [24].

## Conclusions

The complexity of suicide merits the use of multifactorial approaches to effectively deal with the problem. We have shown the rapidity of the uptake of novel methods of gassing in two different populations at three different time periods in the 20^th ^and 21^st ^centuries which in general led to increases in overall suicide rates. Therefore in addition to restricting access to certain methods, national strategies should also focus on surveillance and rapid response to epidemics as well as preventing the spread of new suicide methods from other countries or regions by implementing effective media guidelines and continuously promoting their use.

## Data availability

Data from England and Wales were available on request from the Office for National Statistics. Taiwanese data were from the Department of Health, Taiwan (Department of Health, Taiwan. Cause of death statistics. 2010 [assessed April 7^th ^2010]. Available from http://www.doh.gov.tw/CHT2006/DM/DM2_2.aspx?now_fod_list_no=9521&class_no=440&level_no=2).

## Competing interests

The authors declare that they have no competing interests.

## Authors' contributions

KT contributed to the study design, analysis and interpretation of the paper and drafted the manuscript. SC contributed to the study design, performed the joinpoint regression and helped draft the manuscript. DG conceived the study and helped draft the manuscript. All authors have read and approved the final manuscript.

## Pre-publication history

The pre-publication history for this paper can be accessed here:

http://www.biomedcentral.com/1471-2458/11/314/prepub
